# EXPLORING DELIBERATE PRACTICE TO REDUCE AGE-RELATED PERFORMANCE DIFFERENCES IN A COMPLEX TASK

**DOI:** 10.1093/geroni/igae098.4180

**Published:** 2024-12-31

**Authors:** Michael Prevratil, Neil Charness, Walter Boot

**Affiliations:** Florida State University, Tallahassee, Florida, United States; Florida State University, Tallahassee, Florida, United States; Weill Cornell Medicine, New York, New York, United States

## Abstract

Can age-related differences in complex task performance be overcome with simple behavioral interventions? We monitored differences between older and younger adults’ performance in a complex psychomotor task with the intent of developing a behavioral intervention grounded in principles of deliberate practice. An older (N = 24, M age = 71) and younger (N = 28, M age = 19.1) sample were taught to play a complex videogame called Space Fortress Participants also completed several cognitive tests and a semi-structured interview. The older adults performed significantly worse on Total score (Cohen’s d = 1.45) and three of four subscore metrics (Control, Points, and Speed). Like the differences between men and women (Harwell, Boot, and Ericsson, 2018), score differences between younger and older adults appeared to be driven by strategic and potentially modifiable behaviors. For example, older adults held control inputs for twice as long compared to the younger sample (Cohen’s d = 0.98), a common strategic error. Older adults primarily implemented variants of the “drifter” strategy, letting their ship float uncontrolled through the game environment, while most younger adults implemented the optimal “orbiter” strategy, piloting their ship in a clockwise path around the central fortress. These differences demonstrate likely modifiable aspects of performance that can be improved with targeted behavioral interventions. Findings will be used to develop practice environments and instructions anticipated to greatly reduce or eliminate age-related performance differences.

